# Structural Health Monitoring for Jacket-Type Offshore Wind Turbines: Experimental Proof of Concept

**DOI:** 10.3390/s20071835

**Published:** 2020-03-26

**Authors:** Yolanda Vidal, Gabriela Aquino, Francesc Pozo, José Eligio Moisés Gutiérrez-Arias

**Affiliations:** 1 Control, Modeling, Identification and Applications (CoDAlab), Department of Mathematics, Escola d’Enginyeria de Barcelona Est (EEBE), Universitat Politècnica de Catalunya (UPC), Campus Diagonal-Besòs (CDB), Eduard Maristany, 16, 08019 Barcelona, Spain; francesc.pozo@upc.edu; 2 Facultad de Ciencias de la Electrónica (FCE), Benemérita Universidad Autónoma de Puebla (BUAP), Av. San Claudio y 18 Sur, Ciudad Universitaria, Edificio 1FCE6/202, 72570 Puebla, Mexico; aquino.201006419@gmail.com (G.A.); arigutmses5@gmail.com (J.E.M.G.-A.)

**Keywords:** structural health monitoring, jacket-type, accelerometers, support vector machines, principal component analysis

## Abstract

Structural health monitoring for offshore wind turbines is imperative. Offshore wind energy is progressively attained at greater water depths, beyond 30 m, where jacket foundations are presently the best solution to cope with the harsh environment (extreme sites with poor soil conditions). Structural integrity is of key importance in these underwater structures. In this work, a methodology for the diagnosis of structural damage in jacket-type foundations is stated. The method is based on the criterion that any damage or structural change produces variations in the vibrational response of the structure. Most studies in this area are, primarily, focused on the case of measurable input excitation and vibration response signals. Nevertheless, in this paper it is assumed that the only available excitation, the wind, is not measurable. Therefore, using vibration-response-only accelerometer information, a data-driven approach is developed following the next steps: (i) the wind is simulated as a Gaussian white noise and the accelerometer data are collected; (ii) the data are pre-processed using group-reshape and column-scaling; (iii) principal component analysis is used for both linear dimensionality reduction and feature extraction; finally, (iv) two different machine-learning algorithms, *k* nearest neighbor (*k*-NN) and quadratic-kernel support vector machine (SVM), are tested as classifiers. The overall accuracy is estimated by 5-fold cross-validation. The proposed approach is experimentally validated in a laboratory small-scale structure. The results manifest the reliability of the stated fault diagnosis method being the best performance given by the SVM classifier.

## 1. Introduction

**Figure 1:**
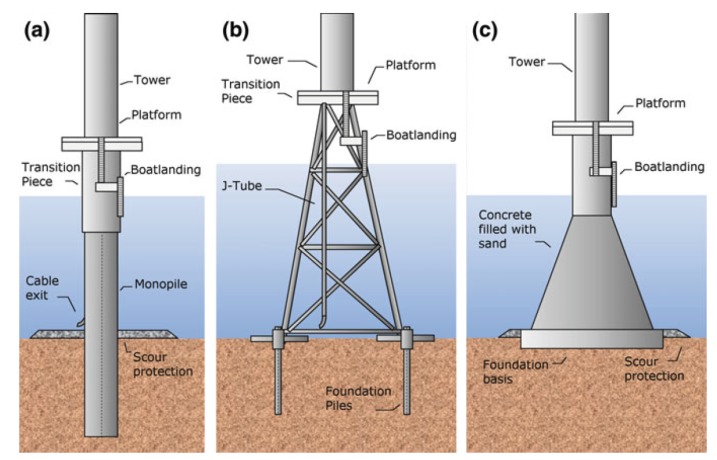
Fixed type WT foundations [1]. Monopile (**a**), jacket (**b**), and gravity-based (**c**).

The potential of offshore wind power is enormous. In offshore wind farms, wind turbines (WTs) are erected with different types of foundations. Monopile foundations are by far the most common foundation (81%). These are quite simple structures anchored directly to the seabed. Gravity foundation systems are very rare (at 5.7% market share) as they involve using a large concrete or steel platform with a diameter of approximately 15 m and a weight of approximately 3000 tons. Finally, jackets are preferred for extreme sites with poor soil conditions as these are foundations with a lattice framework that feature three or four seabed anchoring points, which increases the levels of safety when anchoring the towers; see Figure 1. As said previously, the potential of offshore wind power is enormous. However, it can only be exploited by diminishing operation and maintenance costs. Structural health monitoring (SHM) solutions to provide an early warning of damage are essential to accomplish this objective. Thus, this paper focuses in the problem of damage detection for jacked-type foundations.

In the literature, a lot of methodologies for damage detection can be found, among them the vibration-based methods are one of the most prolific ones, as shown in [2]. Vibration-based SHM methods are data-based approaches employing random excitation and/or vibration response signals (time series), statistical model building, and statistical decision making schemes for inferring the health state of a structure [3]. The interest in these methods has been growing in recent years, due to their simplicity, ease of use, and high effectiveness [4]. However, most studies are, primarily, focused on the case of measurable input excitation and vibration response signals, with only a few recent studies focused on the vibration-response-only case [5], the importance of which stems from the fact that in some applications the excitation cannot be imposed and is often not measurable. This work, aims to contribute in this area of vibration-response-only as the vibration excitation is given by the wind (it cannot be imposed and it is assumed to be unknown).

An overview of SHM systems for various WT components is presented, for example, in [6]. Some important studies that focus specifically on the offshore WT structure are the following. In [7] a review of SHM systems for offshore WTs has been carried out considering the topic as a statistical pattern recognition problem. In [8] health monitoring systems and operational safety evaluation techniques of the offshore wind turbine structure are systematically investigated and summarized. It is noteworthy the work of Mieloszyk et al. [9] where a SHM system is stated based on fiber Bragg grating sensors dedicated to an offshore wind turbine support structure model is presented to detect and localize crack occurrence. It is also remarkable the work of Fritzen et al. [10] where a method for online damage detection/localization is presented accompanied with on field tests of a prototype 5MW plant. In [11] a method for damage localization using finite element model updating is introduced as a subsequent step to a three tier SHM scheme for damage detection. It is also noteworthy the work of Weijtjens et al. [12] related to the foundation SHM of a real offshore monopile WT based on its resonance frequencies where the key problems are the operational and environmental variability of the resonance frequencies of the turbine that potentially conceal any structural change. Another method based on random decrement and neural networks is stated in [13]. A method based on finite element model updating and fuzzy logic is applied on a lab-scale jacket-type platform in [14]. At the lowest level of SHM, the main objective is simply the detection of the presence of damage. In most cases, a model of normality is built [15], and data originating from the structure of interest are tested, usually after some processing, in terms of novelty (when compared to the normal model). Novel data are thus detected and can be considered indicative of damage. Although this process is generally considered less challenging than the full identification of damage, it has a great advantage: it does not need data from damaged states. Finally, in [16], where an experimental testbed similar to the one stated in this work is used, damage detection is accomplished (but not localization or classification) by means of the covariance matrix estimate damage indicator.

This paper contributes a damage detection and localization method (the latter being treated as a classification problem) for a jacket-type WT model by using only acceleration response data. As in [17], it is assumed that the available excitation is the wind, thus the input excitation is not measurable. Hence, the contributed methodology comprises the following steps. First, a Gaussian white noise is used to simulate the wind excitation. Secondly, the data coming from the WT accelerometers are acquired. Thirdly, the raw data are pre-processed using group-reshape (to increase the amount of information contained by each observation) and column-scaling (to simplify the computation of the principal components (PCs)). Fourthly, the PCA is used as a feature selection technique as well as to reduce the dimensionality of the data and the computing time. Finally, the *k*-nearest neighbor (*k*-NN) and the quadratic Support Vector Machine (SVM) classifiers are tested. To estimate their performance, the 5-fold cross-validation technique is used to advise that the SVM has the best performance. The reliability of the proposed method is verified using different bar crack locations in a small-scale structure—an experimental testbed modeling a jacket-type WT.

The structure of the paper is as follows. Section 2 details the experimental laboratory testbed used to validate the proposed approach. Section 3 states the damage detection and classification methodology. The results are presented in Section 4. Finally, the conclusions are drawn in Section 5.

## 2. Experimental Testbed

**Figure 2:**
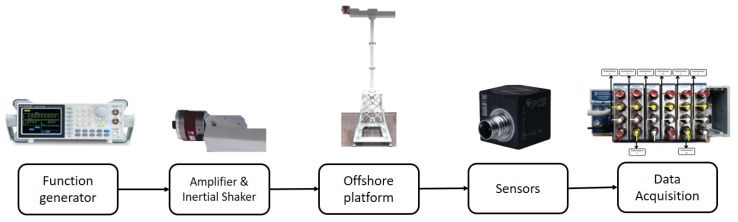
General overview of the experimental testbed.

The general overview of the experimental testbed is given in Figure 2 and explained as follows. The experiment starts with a white noise signal given by the function generator. This signal is amplified and passed to the inertial shaker. This is responsible for generating vibrations (similar to those produced by steady state wind on the blades) to the laboratory tower structure. The shaker is placed in the upper part of the structure, thus simulating the nacelle mass. Finally, the structure is monitored by 8 triaxial accelerometers which are connected to the data acquisition system. The next subsections describe the testbed different steps and instrumentation.

### 2.1. Function Generator

Function generators are signal sources which provide a specifiable voltage applied over a specifiable time. In this work the GW INSTEKAF-2005 model is used. To perform the experimental tests, the white noise signal is selected. Different wind speeds were simulated by multiplying the amplitude of the white noise signal (at the function generator) by the factors 0.5, 1, 2 and 3.

### 2.2. Amplifier and Shaker

**Figure 3:**
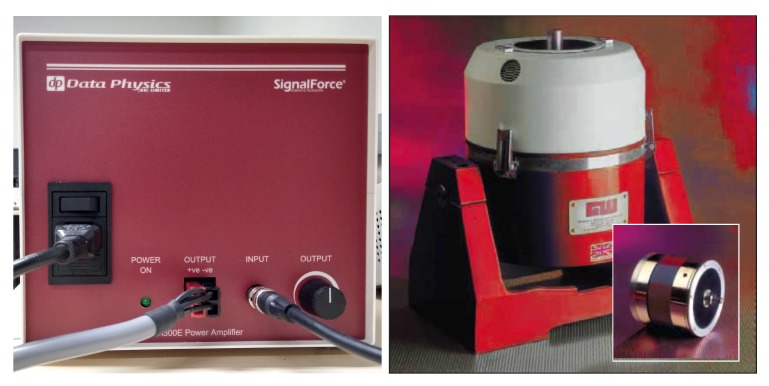
Amplifier model PA300E (left) and inertial shaker IV47 series (right) from Data Physics used in the experimental set-up.

When large structures need to be tested, inertial shakers provide the ideal solution. The central spigot is attached to the structure under test and the body then provides the inertial mass. In this work, the inertial shaker model GW-IV47 from Data Physics is used as well as its PA300E gain control amplifier; see Figure 3. To produce vibrations that simulate the ones obtained when the wind hits the WT blades, the white noise signal given by the function generator is amplified and this electrical signal is applied to the shaker. Thus, the vibration needed to excite the structure is created.

### 2.3. Laboratory Tower Structure and Studied Types of Damage

**Figure 4:**
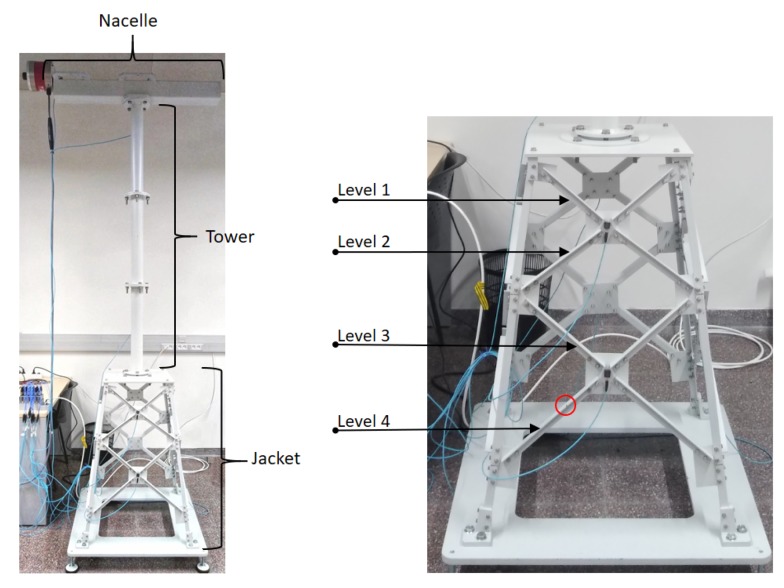
WT scaled offshore fixed jacket-type platform tower model used in the experimental tests (left). Bars (pointed with arrows) where the crack damage is introduced (right); note that in this picture the damage is introduced at level 4 (red circle), but it will be introduced on each level one by one.

The real structure used in this work is 2.7 m high and, as shown in Figure 4 (left), it has three different structural components: nacelle, tower and jacket. The top piece is a 1 m long and 0.6 m wide beam where an inertial shaker is located that simulates the nacelle mass and the environmental effects of the wind over the whole structure. The tower is formed by three tubular sections linked with bolts with a torque of 125 Nm. The jacket is a pyramidal structure formed by several steel bars of different lengths, all of them linked with bolts, with a torque of 12 Nm. The studied damage is introduced in these bars; see Figure 4 (right). In particular, the jacket has four different bar lengths, each one at different levels (depth). Level 1 is where the shortest bars are located, near the water surface. Then, greater depth leads to next levels up to level 4 where the longest bars are situated (near the see bottom). The damage will be introduced, one at a time, at the four different levels, i.e., at four different bars located at level 1, 2, 3 and 4 as illustrated in Figure 4 (right). Fatigue cracks are one of the types of damage found on offshore WT foundations. The probability of detection of a fatigue crack is low for small crack sizes. However, for larger and therefore better detectable fatigue cracks, the crack growth rate accelerates rapidly [18]. Consequently, there is only a small time window for detection and repair of this type of cracks before failure. Thus, in this work a 5 mm crack is considered located at different bars of the jacket structure, one at a time. Please note that in [16] a modal analysis and power spectral density signal processing methods were not able to detect this 5 mm crack located in the substructure using a similar laboratory tower model.

### 2.4. Sensors

**Figure 5:**
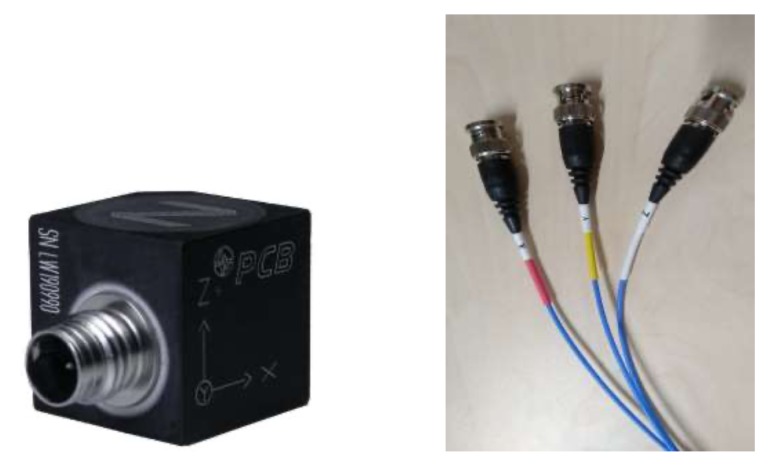
Triaxial accelerometers used in the testbed (PCB Piezotronics, model 356A17).

Eight triaxial accelerometers (model 356A17, PCB Piezotronics) (PCB^®^ manufacturer, Depew, NY, USA) have been used strategically (placed at the tower and jacket by direct adhesive mount) to detect some anomaly in the dynamic behavior of the structure. These are high sensitivity and ceramic shear accelerometers that have a fixed voltage sensitivity, regardless of the type of cable used or its length; and its output signal is low impedance, so it can transmit over long cables in hostile environments without losing signal quality. The device is accompanied by a cable that trifurcates giving an output for each spatial component *x*, *y*, and *z*; see Figure 5. Hence, data from 24 sensors are acquired.

**Figure 6:**
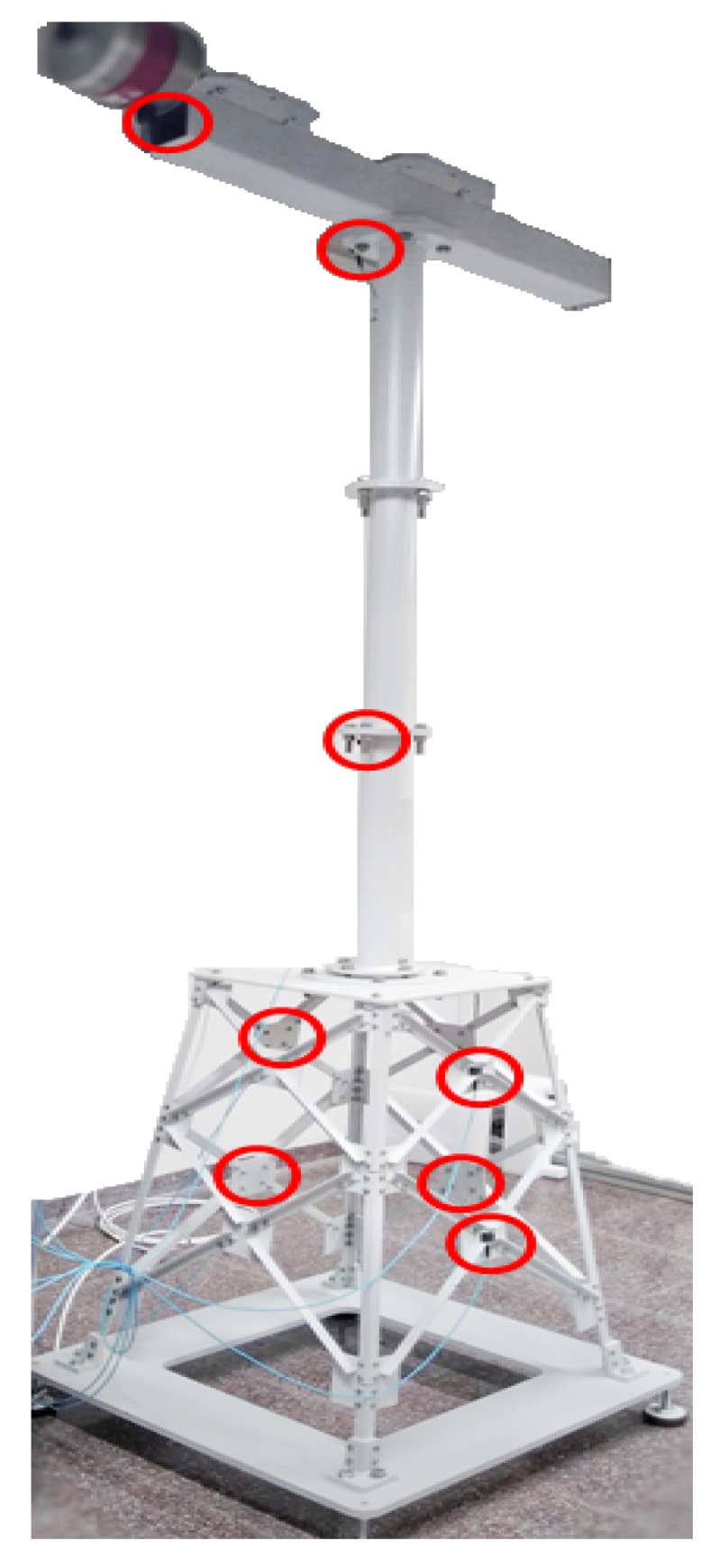
Location of the sensors on the overall structure.

The optimal location of the sensors (see Figure 6) is determined according to the sensor elimination by modal assurance criterion (SEAMAC) ([16], Chapter 3.7, page 53). This is a sensor removal algorithm based on eliminating iteratively, one by one, the degrees of freedom that show a lower impact on MAC matrix values. This iterative process stops when you get a default MAC matrix, high values in the diagonal terms and low values in off-diagonal terms.

### 2.5. Data Acquisition System

**Figure 7:**
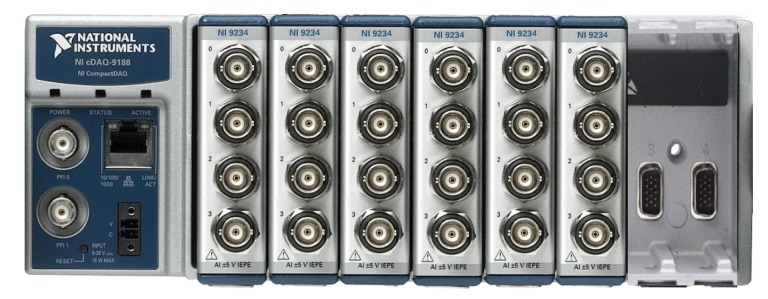
Data Acquisition System (DAQ) used in this work: cDAQ-9188 chassis and six NI-9234 modules from National Instruments.

The data acquisition system is composed by the cDAQ-9188 chassis and six NI-9234 modules from National Instruments™manufacturer (Austin, TX, USA), as shown in Figure 7. The cDAQ-9188 is a CompactDAQ Ethernet chassis, consisting of 8 input slots, and each slot can receive up to 4 different signals. The chassis is capable of controlling timing, synchronization and data transfer between the C Series I / O modules and an external server. The NI-9234 modules can measure signals from integrated electronic piezoelectric sensors (IEPE) and non-IEPE such as accelerometers (used in this work), tachometers and proximity sensors.

## 3. Damage Detection Methodology

### 3.1. Data Collection and Reshape

In this work, data collection and reshape is considered with the goal of combining different response signals (measured by different sensors during multiple observations) into a single and unified view. We will present a method for data fusion, dimensionality reduction and feature extraction using a particular unfolding. We then apply a machine-learning classifier (*k*-NN and SVM are tested) to detect damage or structural changes in incoming collected data. It is clear that the classifiers will play a key role in the damage detection methodology. However, given the three-dimensional nature of the collected information in this paper (time, sensors, experiments), how the data are collected, arranged, scaled, transformed, and reduced may affect the overall performance of the strategy [19]. A similar problem is considered in [20], where the three-dimensional nature of the SHM data comes from location, frequency and time. In that case, tensor analysis is considered to extract the features.

One of the most widely adopted ways to deal with this kind of three-dimensional source of information is the unfolding proposed by Westerhuis et al. [21], where six alternative ways of arranging a 3-dimensional data matrix are proposed. In our case, the combination of the different response signals into a unified view will be represented by a two-dimensional matrix $X=\left(x_{i,j}^{k,l}\right)\in M_{\left(n_{1}+\cdots +n_{E}\right)\times \left(K\cdot L\right)}$ as follows:

(1) $$\begin{array}{cc} X=\left(x_{i,j}^{k,l}\right) & =\left[\begin{array}{cccccccccc} x_{1,1}^{1,1} & \cdots & x_{1,1}^{1,L} & x_{1,1}^{2,1} & \cdots & x_{1,1}^{2,L} & \cdots & x_{1,1}^{K,1} & \cdots & x_{1,1}^{K,L}\\ \vdots & \ddots & \vdots & \vdots & \ddots & \vdots & \ddots & \vdots & \ddots & \vdots \\ x_{n_{1},1}^{1,1} & \cdots & x_{n_{1},1}^{1,L} & x_{n_{1},1}^{2,1} & \cdots & x_{n_{1},1}^{2,L} & \cdots & x_{n_{1},1}^{K,1} & \cdots & x_{n_{1},1}^{K,L}\\ x_{1,2}^{1,1} & \cdots & x_{1,2}^{1,L} & x_{1,2}^{2,1} & \cdots & x_{1,2}^{2,L} & \cdots & x_{1,2}^{K,1} & \cdots & x_{1,2}^{K,L}\\ \vdots & \ddots & \vdots & \vdots & \ddots & \vdots & \ddots & \vdots & \ddots & \vdots \\ x_{n_{2},2}^{1,1} & \cdots & x_{n_{2},2}^{1,L} & x_{n_{2},2}^{2,1} & \cdots & x_{n_{2},2}^{2,L} & \cdots & x_{n_{1},2}^{K,1} & \cdots & x_{n_{2},2}^{K,L}\\ \vdots & \ddots & \vdots & \vdots & \ddots & \vdots & \ddots & \vdots & \ddots & \vdots \\ x_{1,J}^{1,1} & \cdots & x_{1,J}^{1,L} & x_{1,J}^{2,1} & \cdots & x_{1,J}^{2,L} & \cdots & x_{1,J}^{K,1} & \cdots & x_{1,J}^{K,L}\\ \vdots & \ddots & \vdots & \vdots & \ddots & \vdots & \ddots & \vdots & \ddots & \vdots \\ x_{n_{J},J}^{1,1} & \cdots & x_{n_{J},J}^{1,L} & x_{n_{J},J}^{2,1} & \cdots & x_{n_{J},J}^{2,L} & \cdots & x_{n_{J},J}^{K,1} & \cdots & x_{n_{J},J}^{K,L} \end{array}\right]\\ \; & =\left[\begin{array}{c} X_{1}\\ X_{2}\\ \vdots \\ X_{J} \end{array}\right]=\left[\begin{array}{cccc} X^{1} & X^{2} & \cdots & X^{K} \end{array}\right]. \end{array}$$

Matrix X in Equation (1) is presented for a general case, so that the proposed strategy can be easily reproduced. The two subindices *i* and *j* and the two superindices *k* and *l* are related to the experimental trial, structural state, sensor and time instant, respectively. More precisely,

- $i=1,\ldots ,n_{j}$ represents the i−th experimental trial, while $n_{j}$ is the number of observations or experimental trials per structural state;
- j=1,…,J is the structural state that is been measured, while *J* is the quantity of different structural states;
- k=1,…,K indicates the sensor that is measuring, while *K* is the total number of sensors;
- l=1,…,L identifies the time stamp, while *L* is the number of time stamps per experiment.

Please note that matrix X in Equation (1) can also be viewed as the vertical concatenation of *J* matrices $X_{j},\;j=1,\ldots ,J$, where each matrix is associated with a different structural state. Similarly, matrix X can also be considered to be the horizontal concatenation of *K* matrices $X^{k},\;k=1,\ldots ,K$, where each matrix is associated with a different sensor. This horizontal concatenation of matrices can also be viewed as a kind of *group-reshaping*, where we measure a sensor during $n_{j}\cdot L$ time instants (in the j−th structural state), and we finally arrange these $n_{j}\cdot L$ time instants in a $n_{j}\times L$ matrix $X^{j}$. It is noteworthy that by this reshape—that is the key of the selected unfolding proposed by Westerhuis et al. [21]—it is increased the amount of information contained by each observation (row). Moreover, this choice facilitates the study of the variability among samples, because we compile the information related to the sensor measurements and their variations over time.

### 3.2. Column-Scaling and Principal Component Analysis (PCA)

The raw data in matrix X in Equation (1) is scaled for two main reasons: first, to process data that come from different sensors and second, to simplify the computations of the data transformation using PCA [22,23,24,25]. In this work, column-wise scaling (CS) is used. More precisely, each column vector in matrix X is normalized by subtracting the mean of all the elements in the column and by dividing by the standard deviation of the same set of data. Thus, each column of the new scaled matrix, $\overset{˘}{X}$, has a mean of zero and a standard deviation of one.

Recall that before using a classifier, the data must be processed (transformed and reduced) to obtain the most suitable features. In this work, multiway PCA is selected to accomplish this objective. On one hand, the transformation is calculated as a matrix-to-matrix multiplication

$$\begin{array}{c} T=\overset{˘}{X}P, \end{array}$$

where P is the matrix that contains, written as columns, the principal components of matrix $\overset{˘}{X}$. T is a $\left(n_{1}+\cdots +n_{J}\right)\times \left(K\cdot L\right)$ matrix. On the other hand, the dimensionality reduction is performed through the reduced PCA model $P_{\ell }$ that contains, written as columns, the first *ℓ* principal components. More precisely, $T_{\ell }$ is the projection of the scaled matrix $\overset{˘}{X}$ into the vectorial space spanned by the reduced PCA model through the matrix-to-matrix multiplication

$$\begin{array}{c} T_{\ell }=\overset{˘}{X}P_{\ell }. \end{array}$$

Since we have applied column-scaling, the trace of the variance-covariance matrix is equal to K·L. This means that the first *ℓ* principal components retain a proportion of variance given by

$$\begin{array}{c} \frac{\lambda_{1}+\lambda_{2}+\cdots +\lambda_{\ell }}{KL}, \end{array}$$

where $\lambda_{i}$ are the eigenvalues associated with the eigenvectors (principal components) of the variance-covariance matrix, in decreasing order.

### 3.3. Machine-Learning Classifiers

Multi-class classification algorithms are used to categorize the different structural states. In particular, the supervised learning algorithms *k*-NN and SVM are used and its performance compared through different indicators. The *k*-NN classifier is an instance-based learner, i.e., it stores the training data in the memory instead of constructing a model and compares the new test data to closest saved instances to perform the prediction. On the other hand, the SVM classifier constructs a model that is supposed to generalize well. The classifiers are succinctly introduced in the following subsections.

#### 3.3.1. *k*-Nearest Neighbor (*k*-NN)

The *k*-NN algorithm [26,27] stores the training dataset and to make a prediction computes the *k* nearest neighbors to the observation to be categorized and assigns the category of the majority. It only requires tuning one parameter: the number of neighbors, *k*. The main drawback is that as it does not train a model, the algorithm spends more time during the prediction. Please note that we use the same symbol *k* as one of the superindices in the generic element of matrix X in Equation (1), but with a different meaning. We will keep the notation *k*-NN because we think that there is no possibility of ambiguity.

#### 3.3.2. Support Vector Machine (SVM)

It is not the purpose of this paper to give a detailed explanation of the SVM classifier. For the interested reader, an excellent detailed review is given in reference [28]. However, to hand over the background and motivation for the proposed methodology, a summary of the method is given. This recap is based on reference [29].

**Figure 8:**
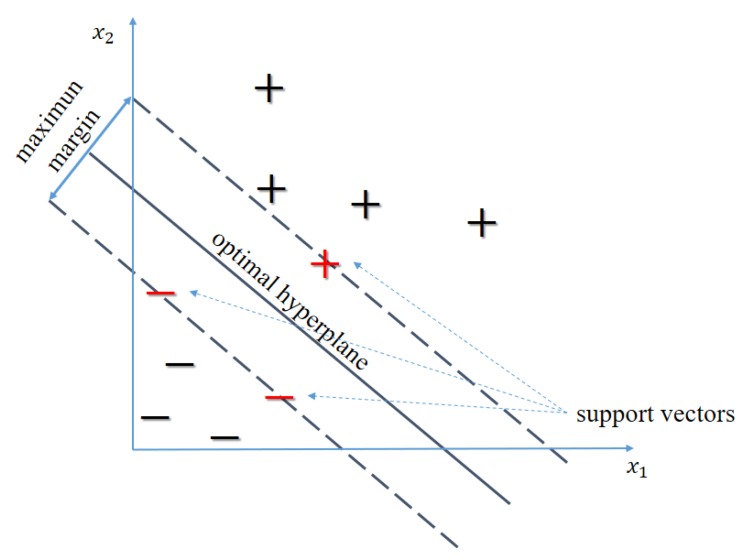
Linear support vector machine (SVM) in a two-dimensional example.

SVM classification is primarily a binary classification technique. Suppose a training set ${\left\{\left(x_{i},y_{i}\right)\right\}}_{i=1}^{N}$ with *d*-dimensional data $x_{i}\in R^{d}$ and their complementary binary label $y_{i}\in \left\{-1,+1\right\}$. Figure 8 shows a two-dimensional example of these type of data where one class is labeled as (+) and the other one as (−). The objective of the SVM is to find the hyperplane with the widest margin to separate both classes; see Figure 8. Conventionally, the hyperplane is given by

(2) $$\begin{array}{c} h\left(x\right)=\omega^{T}x+b, \end{array}$$

where ω is the weight vector and *b* is the bias term. Among all the possible descriptions of the hyperplane, usually the so-called canonical hyperplane is used that satisfies

(3) $$\begin{array}{cc} \omega^{T}x_{+}^{sv}+b & =1, \end{array}$$

(4) $$\begin{array}{cc} \omega^{T}x_{-}^{sv}+b & =-1, \end{array}$$

where $x_{+}^{sv}$ and $x_{-}^{sv}$ illustrate the (+) and (−) training samples closest to the hyperplane (the so-called support vectors); see Figure 8. Maximizing the margin is equivalent to the following minimization problem

(5) $$\begin{array}{c} \min_{\omega ,b}\frac{1}{2}{\left||\omega |\right|}^{2}\;\mathrm{subject}\mathrm{to}\;h\left(x_{i}\right)y_{i}\geq 1,\;i=1,\ldots ,N. \end{array}$$

When the data are not separable by a hyperplane, SVM can use a soft margin, meaning to find a hyperplane that separates many but not all the data. Therefore, the problem is generalized by introducing slack variables, $\varepsilon_{i}$, and a penalty parameter, *C*. Then, the general formulation, for the linear kernel, is,

(6) $$\begin{array}{c} \min_{\omega ,b,\varepsilon_{i}}\frac{1}{2}{\left||\omega |\right|}^{2}+C\sum_{i=1}^{N}\varepsilon_{i}\;\mathrm{subject}\mathrm{to}\;\left\{\begin{array}{c} h\left(x_{i}\right)y_{i}\geq 1-\varepsilon_{i},\;i=1,\ldots ,N;\\ \varepsilon_{i}\geq 0,\;i=1,\ldots ,N. \end{array}\right. \end{array}$$

In this case, using Lagrange multipliers, the problem reads

(7) $$\begin{array}{c} \min_{\alpha_{i}}\left[\sum_{i=1}^{N}\alpha_{i}-\frac{1}{2}\sum_{i=1}^{N}\sum_{j=1}^{N}\alpha_{i}\alpha_{j}y_{i}y_{j}x_{i}^{T}x_{j}\right]\;\mathrm{subject}\mathrm{to}\;\left\{\begin{array}{c} \sum_{i=1}^{N}\alpha_{i}y_{i}=0;\\ 0\leq \alpha_{i}\leq C,\;i=1,\ldots ,N. \end{array}\right. \end{array}$$

The final set of constraints demonstrate why the penalty parameter *C* is called a box constraint, as it retains the values of the Lagrange multipliers in a bounded region.

From Equation (7), it is obvious that optimization depends only on dot products of pairs of samples. Plus, the decision rule depends only on the dot product. Thus, when the classification problem does not have a simple separating hyperplane, even using a soft margin, a transformation to another space can be used, ϕ(·). Indeed, the transformation itself is not needed, but just the dot product (so-called kernel function),

(8) $$\begin{array}{c} K\left(x_{i},x_{j}\right)=\phi \left(x_{i}\right)\phi \left(x_{j}\right). \end{array}$$

**Figure 9:**
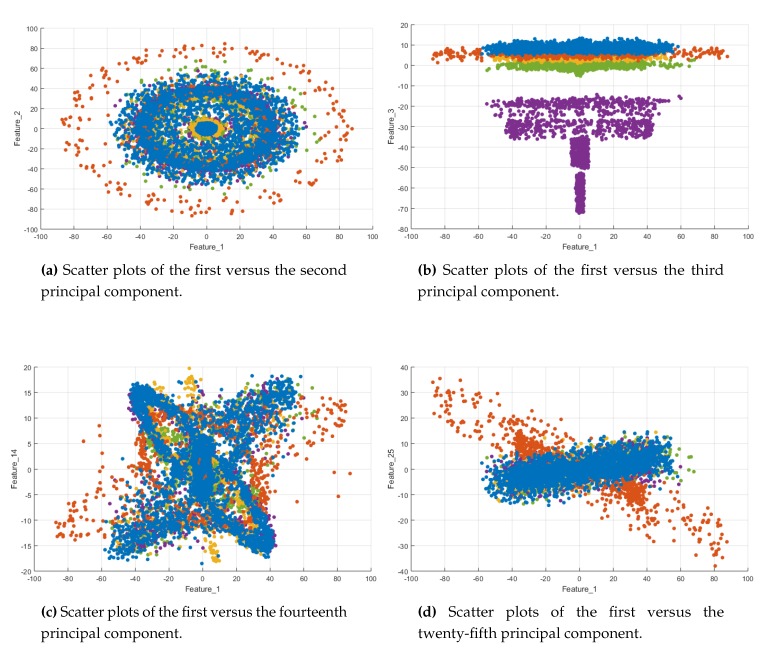
Blue dots represent healthy samples, orange dots represent samples of damage at level 1, yellow dots represent samples of damage at level 2, purple dots represent samples of damage at level 3 and green dots represent samples of damage at level 4.

The kernel function permits the computation of the inner product between the mapped vectors without expressly calculating the mapping. This is known as the *kernel trick* [30]. Different kernels can be used, namely polynomial, hyperbolic tangent, or Gaussian radial basis function. Here, to give an insight and understand why the quadratic kernel is selected in this work, some scatter plots are shown in Figure 9. It can be seen that these plots reveal a quadratic relationship and, particularly, the first versus the second feature scatter plot exposes a concentric circles shape of the data set. Therefore, the quadratic SVM classifier is adopted, i.e., the following polynomial kernel is used

(9) $$K\left(x_{i},x_{j}\right)={\left(1+\frac{1}{\rho^{2}}x_{i}^{T}x_{j}\right)}^{2}$$

where ρ is the so-called kernel-scale parameter; and $x_{i}$ and $x_{j}$ denote here different observations of our data set.

As said previously, SVM classification is a binary classification technique, which must be adapted to cope with multi-classification problems. Two of the most common methods to enable this adaptation include the one-vs-one and one-vs-all approaches. The one-vs-all technique [31] represents the earliest and most common SVM multi-class approach and comprises the division of an *N* class dataset into *N* two-class cases and it designates the class which classifies the test with greatest margin. The one-vs-one strategy [32] comprises constructing a machine for each pair of classes, thus resulting in N(N−1)/2 machines. When this approach is applied to a test point, each classification gives one vote to the winning class and the point is labeled with the class with most votes. The one-vs-one strategy is more computationally demanding since the results of more SVM pairs ought to be computed. In this work, the one-vs-all approach is used.

### 3.4. κ-Fold Cross-Validation

Cross-validation is a technique used to evaluate the results and ensure that they are independent of the partition between training and test data. It comes from the improvement of the holdout method that consists of dividing the sample data into two complementary sets, performing the training in the first subset and validating with the other subset. By the holdout method, the evaluation can depend to a large extent on how the partition between training and test data is performed. Due to this deficiency the concept of cross-validation appears. In the cross-validation of κ iterations, or κ-fold cross-validation, the data is divided into κ subsets. One of the subsets is used as test data and the rest (κ−1) as training data. This process is repeated κ times, and at each iteration a different subset is used as test data and the others as training data. Finally, the arithmetic mean of the results of each iteration is performed to obtain a single result. This method is a more accurate estimate of model prediction performance since it evaluates from κ different combinations of training and test data. In this paper, 5-fold cross-validation is used to estimate the performance of the proposed strategy.

## 4. Results

### 4.1. Experimental Set-Up

As said in Section 2.3, we have considered J=5 different structural states, the healthy WT and the structure with damage located at four different jacket levels. Moreover, a total of $n_{1}+n_{2}+n_{3}+n_{4}+n_{5}=11620$ experimental tests are conducted, which includes the four amplitudes that represent the different wind speed regions (multiplying the amplitude of the white noise signal by the factors 0.5, 1, 2 and 3). In particular:

(i) 1245 tests with the original healthy bar for each amplitude, i.e., $n_{1}=1245\cdot 4=4980$ tests.
(ii) 415 tests with damage located at level 1 for each amplitude, i.e., $n_{2}=415\cdot 4=1660$ tests.
(iii) 415 tests with damage located at level 2 for each amplitude, i.e., $n_{3}=415\cdot 4=1660$ tests.
(iv) 415 tests with damage located at level 3 for each amplitude, i.e., $n_{4}=415\cdot 4=1660$ tests.
(v) 415 tests with damage located at level 4 for each amplitude, i.e., $n_{5}=415\cdot 4=1660$ tests.

For each experimental test, we measure K=24 sensors (see Section 2.4), during L=199 time instants. Since the accelerometers measure at a sampling frequency of about 275 Hz, the time step is Δ=0.003633 seconds, which represents a time window for each experimental test of $\frac{199}{275\;\mathrm{Hz}}=0.7236$ seconds. Please note that this sampling frequency is feasible in an offshore environment using accelerometers; see [8].

Therefore, with the raw matrix X as in Equation (1) where

$$\begin{array}{cc} n_{1} & =4980\\ n_{2} & =1660\\ n_{3} & =1660\\ n_{4} & =1660\\ n_{5} & =1660\\ J & =5\\ L & =199\\ K & =24 \end{array}$$

we apply the column-scaling and we compute the PCA model for the data transformation as detailed in Section 3.2. The extent of the data reduction can be measured as the rate of the number of principal components that retains a predetermined variance with respect to the number of columns in matrix X in Equation (1). For instance:

(i) if we retain 85% of the variance, the first 443 principal components are needed out of K·L=4776 columns. This represents a data reduction (leading to reduced memory requirements) of 90.72%.
(ii) if we retain 90% of the variance, the first 887 principal components are needed. The reduction in this case is 81.43%.
(iii) if we retain 95% of the variance, the first 1770 principal components are needed. This represents a still significant reduction of 62.94%.

Data reduction is of paramount importance in both the training time and the prediction speed. The results in this section are computed and assessed using MATLAB©.

### 4.2. Metrics for Evaluating Classification Models

In classification problems, training data is used to build a model and thus predict the class label for a new sample. To know if the model that we have trained has the best performance according to the problem presented, it is important to evaluate the classification model through metrics such as accuracy, precision, sensitivity and specificity that can be generated from a confusion matrix such as the one shown in Table 1. Regularly, these metrics evaluate binary classification problems.

From this binary confusion matrix:

- True positive (TP) is the number of positive cases that were correctly identified.
- False positive (FP) is the quantity of negative cases that were incorrectly classified as positive.
- True negative (TN) is defined as the sum of negative cases that were correctly classified.
- False negative (FN) is the total of positive cases that were incorrectly classified as negative.

The meaning of *positive* and *negative* may vary from one application to another. For instance, in [23,33], where a 5 MW high-fidelity WT model is considered for fault detection, if a new sample is categorized as positive it means that the current structure is classified as healthy. Otherwise, some kind of fault is present in the WT. In the present work, we follow the same classification with respect to positive and negative.

Table 2 shows the most common metrics for choosing the best solution to a binary classification problem.

As shown in [34], these metrics are easy to calculate and applicable to binary and multi-class classification problems. When the classification problem is multi-class, according to [35,36] the result is the average obtained by adding the result of each class and dividing over the total number of classes. The formulas for calculating these metrics in a multi-class classification model are shown in Table 3.

In a multi-class confusion matrix, the classification results TP, TN, FP and FN can also be considered for each class, as shown in ([35], page 71). Table 4 summarizes a confusion matrix with 5 different classes. When we focus, for instance, on class B (that is, the second class), we can identify four regions:

- the green region is related to the true positive (${\mathrm{TP}}_{2}$);
- the magenta region is related to the false positive (${\mathrm{FP}}_{2}$). More precisely, ${\mathrm{FP}}_{2}$ is the sum of the elements in the second column but BB, i.e., ${\mathrm{FP}}_{2}=\mathrm{AB}+CB+DB+EB$;
- the orange region is related to the false negative (${\mathrm{FN}}_{2}$). More precisely, ${\mathrm{FN}}_{2}$ is the sum of the elements in the second row but BB, i.e., ${\mathrm{FN}}_{2}=BA+BC+BD+BE$; and finally,
- the cyan region is related to the true negative (${\mathrm{TN}}_{2}$). More precisely, ${\mathrm{TN}}_{2}$ is the sum of all the elements of the confusion matrix but the ones in the second row and the second column.

The classification results ${\mathrm{TP}}_{j},{\mathrm{FP}}_{j},{\mathrm{FN}}_{j}$ and ${\mathrm{TN}}_{j}$ for j=1,…,5 that correspond to classes A, B, C, D and E in Table 4, respectively, are computed similarly.

Recall that this paper shows a multiple classification problem that collects data grouped in five different classes: the original healthy bar, damage located at jacket level 1, damage located at jacket level 2, damage located at jacket level 3 and damage located at jacket level 4.

### 4.3. Results of k-NN Classification Method

First, the *k*-NN classifier is tested. The indicators introduced in Section 4.2 are employed to tune the value of the parameter *k*, which is the number of neighbors to be used, and also decide the best variance to be adopted when applying PCA.

**Figure 10:**
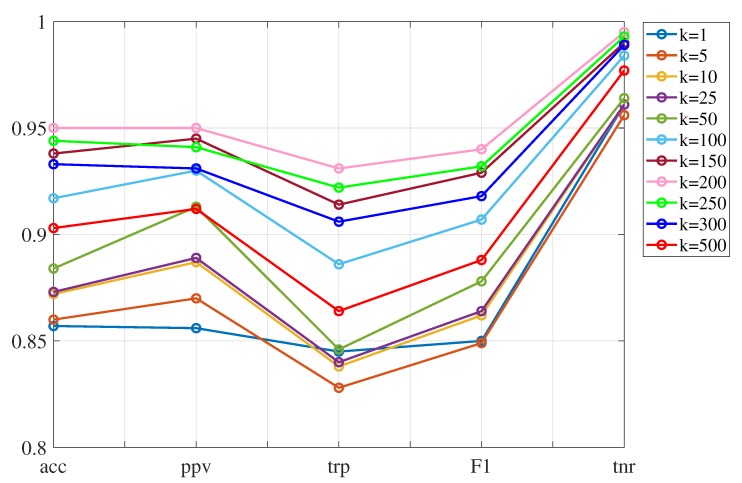
Indicators corresponding to the *k*-NN method using 90% of variance. The case k=200, represented by the pink line, shows the best performance in each of the evaluation indicators.

Table 5 shows the results obtained when working with 85%, 90% and 95% of the variance, and also varying the number of neighbors, *k*. The classifiers with the best performance are highlighted in boldface font. It can be observed that the best classifiers from 90% and 95% of the variance, respectively, have both similar indicators. In this case, working only with 90% of variance is preferred as it will reduce the computational memory requirement. In terms of time, Table 6 shows the training time and prediction speed for each of the classifiers with the best performance. It can be inferred that the classifier using 90% of the variance and k=200 neighbors has the best performance. In particular, for this classifier, Figure 10 graphically shows its performance indicators (pink line) and Table 7 represents its confusion matrix. Regarding Figure 10 it is observed that initially, increasing the number of neighbors leads to better indicators, however when *k* is greater than 200 the performance degrades for all the indicators. With regard to the confusion matrix (Table 7), each row represents the instances in a true class while each column represents the instances in a predicted class (by the classifier). In particular, the first row (and first column) is labeled as 0 and corresponds to the healthy bar. The next labels (for rows and columns) 1, 2, 3, and 4 correspond to bars damage in the corresponding levels of the jacket structure. From this confusion matrix, it can be derived all the aforementioned indicators. In particular, it is noteworthy that an average accuracy of 95%, an average precision (proportion of healthy cases predicted correctly) of 95%, and an average specificity (proportion of faulty cases predicted correctly) of 99.5% are obtained.

### 4.4. Results of SVM Classification Method

In this Section the results of the SVM classification method are presented. As stated in Section 3.3.2, since the scatter plots in Figure 9 reveal a quadratic relationship (particularly the first versus the second principal components), the quadratic SVM classifier is adopted. Therefore, in this case, the hyper-parameters are the box constraint *C*, see (6), and the kernel scale ρ, see (9), that are tuned using the indicators detailed in Section 4.2.

Table 8 summarizes the results obtained when working with 85%, 90% and 95% of the variance. Since the problem we are dealing with seems a separable problem, no changes were found in the performance of the indicators when considering different values of the box constraint *C*. Therefore, C=1 is considered for the rest of the analysis. The cases that present the best results have been highlighted in boldface font. It can be observed that the best classifiers from 85%, 90% and 95% of the variance reach a level of accuracy, precision, recall, F1 score and specificity around 99.8% and 100%. However, the best cases are the ones that consider 85% of the variance and kernel scales ρ=90 and ρ=100. In these cases, the results are almost ideal reaching a specificity value of 100% and the rest of the indicators 99.9%. With respect to the time, Table 9 presents the training time and the prediction speed for each one of the classifiers with the best performance. According to this table, it is observed that the classifier with 85% of the variance and a kernel scale ρ=90 has a higher prediction speed (in terms of observations per second) and a shorter training time. Therefore, this classifier has the best performance. It may seem incongruous that a case with fewer principal components (85%, 443 PCs) behaves better than cases with more principal components (90%, 887 PCs; or even 95%, 1770). However, this can occur, since the last principal components usually collect the noise present in the measurements.

**Figure 11:**
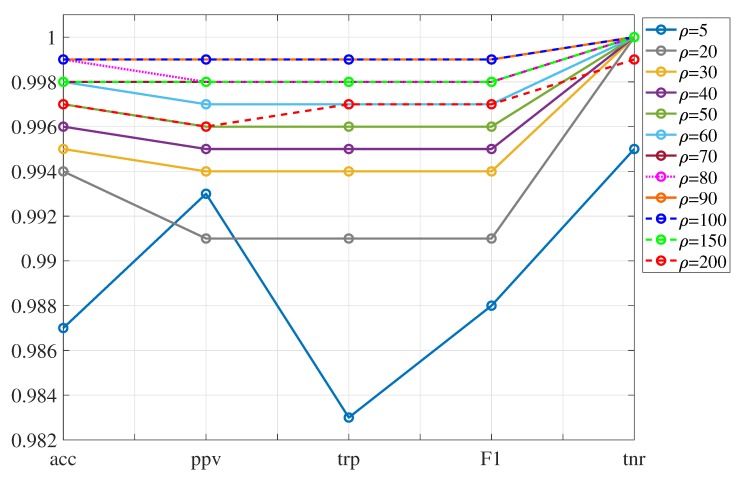
Indicators to evaluate the SVM classification model using 85% of variance. The cases with the best performance are ρ=90 and ρ=100 that are represented by the overlapped orange (solid) line and blue (dotted) line, respectively. The graph omits ρ=300 since the results obtained are much worse and would be out of the y-axis scale used in this graph.

Figure 11 graphically shows the magnitude of the indicators in Table 3 with respect to the kernel scale ρ for the case with 85% of the variance. It can be seen that increasing the kernel scale from ρ=5 (solid light blue) onwards improves the overall performance of the classification method up to a certain limit. The performance degradation appears for values greater than ρ=100.

The confusion matrix for the best performing classifier (85% variance and kernel scale ρ=90) is represented in Table 10. From this confusion matrix and according to the indicators of evaluation, we obtain an average accuracy of 99.9%, an average precision of 99.9% and an average specificity of 100%.

## 5. Conclusions

In this work, a methodology has been stated for damage detection and localization on a laboratory-scale WT with jacket-type foundation. In particular, a crack damage is studied in four different locations of the jacket foundation. The main conclusions stressed from this work are the following:

(i) A vibration-response-only methodology has been conceived and a satisfactory experimental proof of concept has been conducted. However, future work is needed to validate the technology in a more realistic environment that takes into account the varying environmental and operational conditions.
(ii) The contribution of this work resides in how three-dimensional data (coming from different time, sensors, and experiments) is collected, arranged, scaled, transformed, and dimension reduced following a general framework stated in Section 3.1 and Section 3.2, and afterwards particularized for the specific application that concerns us in Section 4.1.
(iii) The damage detection and localization methodology with the quadratic SVM classifier, kernel scale ρ=90, box constraint C=1, and 443 principal components (85% of variance kept) has a very close to ideal performance, achieving in all indicators a result equal o higher to 99.99% with a very fast prediction speed (1700 obs/sec) and short training time (76 sec).

Finally, it is important to note that environmental and operational conditions (EOC) play an important role when dealing with long term monitoring, because they can complicate damage detection. Large variations in EOCs make EOC monitoring almost as important as structural monitoring itself. Therefore, its influence should be compensated. Several methods for EOC compensation for WTs have been developed to make SHM possible. For example, in [37] affinity propagation clustering is used to delineate data into WT groups of similar EOC. In [38] covariance-driven stochastic subspace identification is used. Finally, in [39,40] fuzzy classification techniques are used for EOC compensation. However, as noted previously, this work is an experimental proof of concept and EOC compensation is left as future work using pattern recognition techniques in a more realistic environment. 

## Figures and Tables

**Table 1:** Binary confusion matrix.

		Predicted Class	
		Positive	Negative
**Actual class**	**Positive**	True positive (TP)	False negative (FN)
	**Negative**	False positive (FP)	True negative (TN)

**Table 2:** Metrics for evaluating binary classification models.

Metric	Formula	Description
Accuracy	$\mathrm{acc}=\frac{\mathrm{TP}+\mathrm{TN}}{\mathrm{TP}+\mathrm{FP}+\mathrm{FN}+\mathrm{TN}}$	It is the number of correct predictions made by the model according to the total number of records. The best accuracy is 100%, which indicates that all predictions are correct. Accuracy alone does not tell the full story when working with a class-imbalanced data set.
Precision	$\mathrm{ppv}=\frac{\mathrm{TP}}{\mathrm{TP}+\mathrm{FP}}$	This parameter evaluates the data by its performance of *positive* predictions, in other words, it is the proportion of positive cases that have been correctly predicted.
Sensibility/Recall	$\mathrm{tpr}=\frac{\mathrm{TP}}{\mathrm{TP}+\mathrm{FN}}$	This parameter calculates the fraction of the positive cases that our model has been able to identify as positive (true positive).
F1-Score	$F1=2\frac{\mathrm{ppv}\;\times \;\mathrm{tpr}}{\mathrm{ppv}\;+\mathrm{tpr}}$	It is defined as the harmonic mean of precision and sensitivity. A F1 score reaches its best value at 1 (accuracy and perfect sensitivity) and worse at 0.
Specificity	$\mathrm{tnr}=\frac{\mathrm{TN}}{\mathrm{TN}+\mathrm{FP}}$	The specificity or true negative rate is calculated as the proportion of correct negative predictions divided by the total number of cases that are classified as negative. Specificity is the exact opposite of sensitivity, the proportion of negative cases predicted correctly.

**Table 3:** Metrics for evaluating multi-class classification models, where *j* refers to the individual class and *J* is the total number of classes.

Metric	Formula
Average Accuracy ($\bar{acc}$)	$\frac{1}{J}\sum_{j=1}^{J}\frac{{\mathrm{TP}}_{j}+{\mathrm{TN}}_{j}}{{\mathrm{TP}}_{j}+{\mathrm{FP}}_{j}+{\mathrm{FN}}_{j}+{\mathrm{TN}}_{j}}$
Average Precision ($\bar{ppv}$)	$\frac{1}{J}\sum_{j=1}^{J}\frac{{\mathrm{TP}}_{j}}{{\mathrm{TP}}_{j}+{\mathrm{FP}}_{j}}$
Average Sensibility/Recall ($\bar{tpr}$)	$\frac{1}{J}\sum_{j=1}^{J}\frac{{\mathrm{TP}}_{j}}{{\mathrm{TP}}_{j}+{\mathrm{FN}}_{j}}$
Average F1-Score ($\bar{F1}$)	$2\frac{\bar{ppv}\times \bar{tpr}}{\bar{ppv}+\bar{tpr}}$
Average Specificity ($\bar{tnr}$)	$\frac{1}{J}\sum_{j=1}^{J}\frac{{\mathrm{TN}}_{j}}{{\mathrm{TN}}_{j}+{\mathrm{FP}}_{j}}$

**Table 4:** Multi-class confusion matrix. In this case, the confusion matrix has five classes.

		Predicted Class				
		Class A	Class B	Class C	Class D	Class E
**Actual class**	**Class A**	AA	AB	AC	AD	AE
	**Class B**	BA	BB	BC	BD	BE
	**Class C**	CA	CB	CC	CD	CE
	**Class D**	DA	DB	DC	DD	DE
	**Class E**	EA	EB	EC	ED	EE

**Table 5:** Evaluation indicators for the *k*-NN method using different percentages of variance and different number of nearest neighbors (*k*). The best classifiers for each variance are highlighted using a boldface font.

Variance	Number of PCs	Neighbors *k*	Accuracy $\bar{acc}$	Precision $\bar{ppv}$	Recall $\bar{tpr}$	F1 score $\bar{F1}$	Specificity $\bar{tnr}$
85%	443	1	0.857	0.860	0.846	0.853	0.950
		5	0.856	0.869	0.82	0.844	0.953
		10	0.867	0.891	0.827	0.858	0.956
		25	0.868	0.898	0.828	0.862	0.955
		50	0.874	0.912	0.831	0.87	0.958
		100	0.911	0.928	0.879	0.903	0.981
		150	0.933	0.942	0.908	0.925	0.988
		200	**0.946**	**0.947**	**0.924**	**0.936**	**0.993**
		250	0.940	0.938	0.917	0.927	0.991
		300	0.930	0.929	0.903	0.915	0.988
		500	0.899	0.909	0.859	0.884	0.976
90%	887	1	0.857	0.856	0.845	0.85	0.961
		5	0.860	0.870	0.828	0.849	0.956
		10	0.872	0.887	0.838	0.862	0.961
		25	0.873	0.889	0.840	0.864	0.961
		50	0.884	0.913	0.846	0.878	0.964
		100	0.917	0.930	0.886	0.907	0.984
		150	0.938	0.945	0.914	0.929	0.990
		200	**0.950**	**0.950**	**0.931**	**0.940**	**0.995**
		250	0.944	0.941	0.922	0.932	0.993
		300	0.933	0.931	0.906	0.918	0.989
		500	0.903	0.912	0.864	0.888	0.977
95%	1770	1	0.854	0.853	0.842	0.847	0.960
		5	0.858	0.865	0.829	0.847	0.956
		10	0.872	0.885	0.841	0.862	0.962
		25	0.873	0.888	0.841	0.864	0.962
		50	0.885	0.911	0.848	0.879	0.965
		100	0.918	0.930	0.888	0.908	0.985
		150	0.938	0.945	0.915	0.930	0.990
		200	**0.950**	**0.950**	**0.930**	**0.940**	**0.995**
		250	0.945	0.942	0.923	0.932	0.993
		300	0.934	0.931	0.908	0.919	0.989
		500	0.904	0.913	0.866	0.889	0.978

**Table 6:** Prediction speed and training time results for the best performance classifiers of each variance (85%, 90% and 95%) using the *k*-NN classification method on a 3GHz Intel Core i7, 16 GB RAM computer. The best classifier in terms of accuracy is highlighted using a boldface font.

Variance	Accuracy	Prediction Speed (obs/s)	Training Time (s)
85%	94.6%	210	329
**90%**	**95.0%**	**110**	**638**
95%	95.0%	55	1251

**Table 7:** Confusion matrix for the *k*-NN algorithm with k=200 neighbors. The obtained accuracy is 95%. Label 0 corresponds to the healthy state, and labels 1, 2, 3 and 4 correspond to the damage state located at the corresponding level. In this matrix, each row represents the instances in a true class while each column represents the instances in a predicted class. An empty blank square means 0%.

	0	1	2	3	4
0	>99%		<1%		
1	<1%	98%	2%		
2	7%		93%		<1%
3				75%	25%
4	1%		<1%		99%

**Table 8:** Evaluation indicators for the SVM model using different percentages of variance and different values for ρ (kernel scale). The best values for each indicator are highlighted using a boldface font.

Variance/ Number of Components	Box Constraint C	Kernel Scale ρ	Accuracy $\bar{acc}$	Precision $\bar{ppv}$	Recall $\bar{tpr}$	F1 Score $\bar{F1}$	Specificity $\bar{tnr}$
85%/443	1	5	0.987	0.993	0.983	0.988	0.995
		20	0.994	0.991	0.991	0.991	**1.000**
		30	0.995	0.994	0.994	0.994	**1.000**
		40	0.996	0.995	0.995	0.995	**1.000**
		50	0.997	0.996	0.996	0.996	**1.000**
		60	0.998	0.997	0.997	0.997	**1.000**
		70	0.998	0.998	0.998	0.998	**1.000**
		80	**0.999**	0.998	0.998	0.998	**1.000**
		90	**0.999**	**0.999**	**0.999**	**0.999**	**1.000**
		100	**0.999**	**0.999**	**0.999**	**0.999**	**1.000**
		150	0.998	0.998	0.998	0.998	**1.000**
		200	0.997	0.996	0.997	0.997	0.999
		300	0.882	0.926	0.835	0.878	0.954
90%/887	1	5	0.987	0.993	0.982	0.988	0.995
		20	0.991	0.988	0.988	0.988	**1.000**
		30	0.995	0.993	0.993	0.993	**1.000**
		40	0.996	0.995	0.995	0.995	**1.000**
		50	0.997	0.997	0.997	0.997	**1.000**
		60	**0.998**	0.997	0.997	0.997	**1.000**
		70	**0.998**	0.997	0.997	0.997	**1.000**
		80	**0.998**	0.997	0.997	0.997	**1.000**
		90	**0.998**	**0.998**	**0.998**	**0.998**	**1.000**
		100	**0.998**	**0.998**	**0.998**	**0.998**	**1.000**
		150	**0.998**	**0.998**	**0.998**	**0.998**	0.999
		200	0.997	0.996	0.996	0.996	0.999
		300	0.883	0.927	0.837	0.880	0.955
95%/1770	1	5	0.986	0.993	0.981	0.987	0.994
		20	0.990	0.987	0.986	0.986	**1.000**
		30	0.994	0.992	0.992	0.992	**1.000**
		40	0.996	0.994	0.994	0.994	**1.000**
		50	0.997	0.996	0.996	0.996	**1.000**
		60	0.997	0.997	0.997	0.997	**1.000**
		70	**0.998**	0.997	0.997	0.997	**1.000**
		80	**0.998**	0.997	**0.998**	**0.998**	**1.000**
		90	**0.998**	**0.998**	**0.998**	**0.998**	**1.000**
		100	**0.998**	0.997	**0.998**	**0.998**	**1.000**
		150	**0.998**	0.997	**0.998**	0.997	0.999
		200	0.996	0.996	0.996	0.996	0.999
		300	0.997	0.997	0.997	0.997	0.999

**Table 9:** Prediction speed and training time results for the best performance cases of each variance (85%, 90% and 95%) using the SVM classification method. The best classifier in terms of accuracy and computational cost is highlighted using a boldface font.

Variance	Kernel Scale ρ	Accuracy	Prediction Speed (obs/sec)	Training Time (sec)
**85%**	**90**	**0.999**	**1700**	**76**
	100	0.999	1600	79
90%	90	0.998	320	256
	100	0.998	580	364
95%	90	0.998	100	676

**Table 10:** Confusion matrix of the SVM model with ρ=90 and C=1, we get an accuracy of 99.9%. Label 0 corresponds to healthy, label 1 corresponds to level 1, label 2 corresponds to level 2, label 3 level 3 and label 4 corresponds to level 4. In this matrix, each row represents the instances in a true class while each column represents the instances in a predicted class. An empty blank square means 0%.

	0	1	2	3	4
0	>99%	<1%	<1%		
1	<1%	99%	<1%		
2		<1%	99%		
3				>99%	<1%
4					100%

## Abbreviations

The following abbreviations are used in this manuscript:

CS	column-scaling
FN	false negative
FP	false positive
*k*-NN	*k* nearest neighbors
IEPE	integrated electronic piezoelectric sensors
MAC	modal assurance criterion
PC	principal component
PCA	principal component analysis
SHM	structural health monitoring
SVM	support vector machine
TN	true negative
TP	true positive
WT	wind turbine
